# Prevalence of *Entamoeba histolytica* and other enteral parasitic diseases in the metropolitan region of Belo Horizonte, Brazil. A cross-sectional study

**DOI:** 10.1590/1516-3180.2018.0036170418

**Published:** 2018-08-13

**Authors:** Juliana de Oliveira Costa, José Adão Resende, Frederico Ferreira Gil, Joseph Fabiano Guimarães Santos, Maria Aparecida Gomes

**Affiliations:** I MPH, PharmD. Pharmacist, Department of Social and Preventive Medicine, Postgraduate Program in Public Health, Universidade Federal de Minas Gerais (UFMG), Belo Horizonte (MG), Brazil.; II Clinical analysis technician, Hermes Pardini Laboratory, Belo Horizonte (MG), Brazil.; III PhD. Nurse, Department of Parasitology, Universidade Federal de Minas Gerais (UFMG), Belo Horizonte (MG), Brazil.; IV MD, PhD. Physician, Hospital Universitário Lucas Machado, Fundação Educacional Lucas Machado, Belo Horizonte (MG), Brazil.; V PharmD, PhD. Full Professor, Department of Parasitology, Universidade Federal de Minas Gerais (UFMG), Belo Horizonte (MG), Brazil.

**Keywords:** Parasitic diseases, Entamoeba histolytica, Prevalence, Brazil

## Abstract

**BACKGROUND:**

Enteral parasitic diseases are a public health problem in nations with low economic development and in settings with poor sanitation. Amebiasis is the second most frequent form of parasitosis, with a high burden of disease. Knowledge of the prevalence of enteroparasitoses in a given region is useful for planning clinical decision-making. Thus, the aim of this study was to estimate the prevalence of enteral parasitic diseases, especially amebiasis, through analysis on stool samples from public and private laboratories in a metropolitan area in southeastern Brazil.

**DESIGN AND SETTING:**

Cross-sectional study conducted in the metropolitan region of Belo Horizonte, Brazil.

**METHODS:**

We evaluated 6,289 fecal samples from one private and one public laboratory. The samples were concentrated by means of spontaneous sedimentation, and those that were positive for *Entamoeba histolytica* or *Entamoeba dispar* in optical microscopy analyses were processed to obtain deoxyribonucleic acid, with subsequent identification through the polymerase chain reaction.

**RESULTS:**

Among the stool samples, 942 (15.0%) had parasitic infections; 73 (1.2%) of these were helminthic infections and 847 (13.5%) were protozoan infections, caused mainly by *Escherichia coli* (6.0%), *Endolimax nana* (5.2%) and *Giardia lamblia* (1.2%). Infections due to *Entamoeba histolytica* or *Entamoeba dispar* occurred in 36 samples (0.6%) and the polymerase chain reaction revealed five (13.9%) as *Entamoeba histolytica*.

**CONCLUSION:**

The prevalence of enteral parasitic diseases is high in the metropolitan region of Belo Horizonte, although amebiasis may not be a problem.

## INTRODUCTION

Intestinal parasites affect approximately 3.5 billion people worldwide and are a public health problem, especially in developing countries, where almost one-third of the population live in conditions favorable to their dissemination.^1^^,^^2^ Worldwide, amebiasis is the second most frequent parasitic disease, causing around 100,000 deaths each year and contributing towards the high global burden of diarrhea, notably in regions with low economic development and settings with poor sanitation.^2^^,^^3^^,^^4^ Intestinal parasites are responsible for high levels of morbidity. The impact of intestinal nematode infections has been estimated to exceed five million disability-adjusted life years (DALYs), while amebiasis alone accounts for more than two million DALYs.^5^

The prevalence of enteroparasitosis in Brazil can reach over 60%^6^ and varies across regions according to educational, economic and social levels, the population agglomeration index, sanitary conditions, soil contamination levels, contamination of water and food supplies and the virulence capacity of parasites.^7^^,^^8^ These variations often make comparisons inadequate.

The prevalence of amebiasis ranges from 3.4% to 40.0%,^4^^,^^9^ but these estimates may be imprecise because of the existence of *Entamoeba dispar*, which is morphologically indistinguishable from *Entamoeba histolytica.*^10^ Differentiation between these species is very important for epidemiological and clinical purposes, such as selection of adequate therapy and investigation of other causes for gastrointestinal symptoms.^10^ Brazilian studies aiming to characterize these two species in the general population are scarce,^4^^,^^11^^,^^12^^,^^13^ and other studies have been conducted in specific populations such as schoolchildren^14^ or regions without access to water,^15^ or have compared the diagnostic methods available.^16^

The objective of the present study was to estimate the prevalence of enteral parasitic diseases in the general population using data from both public and private laboratories in the city of Belo Horizonte, Brazil, using molecular methods to distinguish between *Entamoeba histolytica* and *Entamoeba dispar* infections.

## METHODS

### Study design, setting, sampling and ethics evaluation 

This was a cross-sectional study conducted in the metropolitan region of Belo Horizonte, Brazil. This study was approved by the ethics committee of the municipality of Belo Horizonte under the protocol number 021/2007.

Belo Horizonte, the capital of the state of Minas Gerais, is located in the southeastern region of Brazil. In the year 2008, when this study was conducted, Belo Horizonte had 2,399,920 inhabitants and had a human development index of 0.810, which is classified as high.^17^

Considering the population size of Belo Horizonte, an anticipated prevalence of *Entamoeba histolytica* or *Entamoeba dispar* of 20%, at a significance level of 5%, with 20% data loss, a sample of 296 patients would be required to estimate the prevalence of amebiasis in the city. However, due to the ease of access to the results from examinations, we included a total of 6,289 samples.

### Stool sample origin

We included samples from the largest private laboratory in Belo Horizonte and from the city’s municipal laboratory, from May to June 2008. The private laboratory processes approximately 800 fecal samples/day, from a wide variety of regions of Belo Horizonte and from adjacent regions. Because of the large number of stool examinations performed daily, samples from one week were included in the study.

The laboratory accredited to the network of clinical analysis laboratories of the municipality of Belo Horizonte attends communities on the outskirts of the city. We selected four low-income communities distributed in the northern, eastern, northeastern and central-southern regions. We chose one public and one private laboratory to evaluate possible differences in the prevalence of enteroparasitosis regarding socioeconomic conditions of their users. The network of public laboratories processes approximately 60 fecal samples/day and, therefore, samples covering one month were included in the study.

All fecal samples were conditioned at 4 °C and concentrated by means of spontaneous sedimentation not more than 24 hours after their receipt for analysis. All positive samples for *Entamoeba histolytica* and *Entamoeba dispar* were stored at 4 °C until they were sent to the amebiasis laboratory of the Federal University of Minas Gerais (Universidade Federal de Minas Gerais, UFMG) for identification by means of the polymerase chain reaction.

### Method for deoxyribonucleic acid extraction

Deoxyribonucleic acid (DNA) was isolated by means of alkaline lysis in accordance with methodology that had previously been described,^18^ with some modifications to minimize inhibition by fecal constituents. Polyvinylpolypyrrolidone (200 µl) was added to 200 µl of fecal sediment, and this mixture was incubated at 100 °C for 10 minutes. Lysis buffer (50 mM glucose, 25 mM Tris-HCl and 10 mM EDTA) was added to this material, with five minutes of incubation at room temperature. A solution of 0.2 mM NaOH was then added to the mixture together with 1% sodium dodecyl sulfate, and the mixture was then frozen in liquid N_2_ and thawed, three times. Then 300 µl of 7.5 mM ammonium acetate was added, and this mixture was incubated in ice for 20 minutes, followed by centrifugation at 12,500 g for 5 minutes. The supernatant was then precipitated with 0.6 volumes of isopropanol. The purified deoxyribonucleic acid was resuspended in 20 μl of TE buffer (10 mM Tris-HCl and 1 mM EDTA).

### Polymerase chain reaction

The polymerase chain reaction (PCR) target was a 300 bp ribosomal deoxyribonucleic acid segment that was amplified in *Entamoeba histolytica* and *Entamoeba dispar* in distinct reactions using different direct primers (Eh GTACAAAATGGCCAATTCATTC and Ed GTACAAAGTGGCCAATTTATG) and the same reverse primer (Eh/Ed GAATTGATTTTACTCAACTCTAG). The target of this polymerase chain reaction was a gene fragment of a small ribosomal subunit of ribonucleic acid. This target was chosen because it was present at a rate of around 200 copies per trophozoite,^19^ thus providing greater sensitivity for making the diagnosis. The differences in the sequence of this gene between *Entamoeba histolytica* and *Entamoeba dispar* have made it possible to design specific primers for these two species.^20^ The reaction took place over 50 cycles, with an annealing temperature of 57 °C and individual cycles of 30 seconds each.

### Variables and statistical analysis 

The numbers of positive results were described in terms of frequencies and proportions according to the type of parasite and were stratified according to the origin of the fecal sample, i.e. private or public laboratory. The chi-square test or Fisher’s exact test was used, as appropriate, to compare proportions of infections between the laboratories. We considered differences with a P-value < 0.05 to be statistically significant. We used the OpenEpi v.3.01 software for the statistical analysis.

## RESULTS

In the private laboratory, 5,695 stool examinations were evaluated during the data collection. The participants’ mean age was 42.0 ± 34.0 years. In the public laboratory, 594 stool examinations were evaluated, and the patients’ mean age was 29.6 ± 20.1 years.

The private laboratory registered 772 positive tests for enteral parasitic diseases (13.5%), while the public laboratory identified 170 positive samples (28.6%), and this difference in the proportions of positive samples was statistically significant (P < 0.001). The proportion of infections according to the type of parasite was also statistically lower in the private laboratory, such that helminthic infections accounted for 0.8% of the parasites, compared with 4.9% of the samples processed in the public laboratory (P < 0.001). Protozoal infections occurred in 12.8% of the samples at the private laboratory and in 20.0% of those at the public laboratory, and this difference was statistically significant (P < 0.001).

The distribution of intestinal parasites according to the etiological agent and the laboratory is described in Table 1. In general, the same helminths presented greatest prevalence in both places, but with differences in magnitude. In the private laboratory, larvae of *Strongyloides stercoralis* and eggs of *Schistosoma mansoni* were the most prevalent forms (0.2%), followed by eggs of *Enterobius vermicularis, Ascaris lumbricoides* and *Taenia* (0.1%). In the public laboratory, eggs of *Ascaris lumbricoides* were the most prevalent (2.0%), followed by larvae of *Strongyloides stercoralis* (1.0%), eggs of *Trichuris trichiura* (0.5%), *Schistosoma mansoni* (0.5%), *Enterobius vermicularis* (0.3%), Ancylostomatidae and *Taenia* (0.2%). The prevalences of eggs of *Ascaris lumbricoides, Trichuris trichiura* and *Strongyloides stercoralis* larvae were statistically higher in the public laboratory (P < 0.05).

**Table 1:** Prevalence of intestinal parasites in Belo Horizonte, according to the type of laboratory (n = 6,289)

Parasite	Private laboratory (n = 5,695) n (%)	Public laboratory (n = 594) n (%)	Total n (%)	**P*-*value**
*Blastocystis hominis*	6 (0.1)	0 (0.0)	6 (0.1)	> 0.999*
*Endolimax nana*	305 (5.4)	21 (3.5)	326 (5.2)	0.071
*Escherichia coli*	321 (5.6)	56 (9.4)	377 (6.0)	0.000
*Entamoeba histolytica/ Entamoeba dispar*	23 (0.4)	13 (2.2)	36 (0.6)	0.000*
*Giardia lamblia*	52 (0.9)	26 (4.4)	78 (1.2)	< 0.000
*Iodamoeba bütschlii*	13 (0.2)	3 (0.5)	16 (0.3)	0.374*
*E. hartmanni*	-	0 (0.0)	0 (0.0)	-
*Trichuris trichiura*	1 (0.0)	3 (0.5)	4 (0.1)	0.006*
*Ascaris lumbricoides*	4 (0.1)	12 (2.0)	16 (0.3)	< 0.000*
*Strongyloides stercoralis*	13 (0.2)	6 (1.0)	19 (0.3)	0.012*
Hookworm	2 (0.0)	1 (0.2)	3 (0.0)	0.514
*Cryptosporidium*	-	0 (0.0)	0 (0.0)	-
*Taenia*	3 (0.1)	1 (0.2)	4 (0.1)	0.655*
*Enterobius vermicularis*	6 (0.1)	2 (0.3)	8 (0.1)	0.341*
*Hymenolepis*	1 (0.0)	1 (0.2)	2 (0.0)	0.360*
*Schistosoma mansoni*	10 (0.2)	3 (0.5)	13 (0.2)	0.235*

*Fisher’s exact test; - = data not available.

The most prevalent protozoon found in both laboratories was *Entamoeba coli*, which accounted for 5.6% and 9.4% of the cases in the private and public laboratories, respectively (P < 0.01). *Endolimax nana* ranked second in the private laboratory and third in the public laboratory, without a statistically significant difference between the groups (5.4% versus 3.5%; P = 0.07). In contrast, the prevalence of *Giardia lamblia* was higher in the public laboratory (0.9 versus 4.4%; P < 0.001), as also was the prevalence of *Entamoeba histolytica* or *Entamoeba dispar* (0.4 versus 2.2%; P < 0.001).

Polymerase chain reaction analysis on the samples that had been found to be positive through optical microscopy identified five cases of *Entamoeba histolytica* in the population studied, which was a prevalence of 0.08%. The proportion of cases due to *Entamoeba histolytica* in the *Entamoeba histolytica/Entamoeba dispar* complex was 15.0% (3/23) in the private laboratory and 15.4% (2/13) in the public laboratory, thus totaling 13.9% of the cases.

## DISCUSSION

In this study, the prevalence of intestinal parasites was 15.0%, through optical microscopy. A similar rate was reported in a previous study in Belo Horizonte, and that study also highlighted high prevalence among members of the families of infected individuals.^21^ Considering that a safe water supply was available in the region studied, other socioeconomic, behavioral and lifestyle characteristics may have played an important role in the transmission of intestinal parasites, such as consumption of uncooked food, low levels of personal hygiene and lack of usage of footwear.^6^^,^^21^

The proportion of the samples that was identified as infected at the public laboratory was almost twice what was identified at the private laboratory. Intestinal parasites are more common among low-income individuals with lower schooling levels.^22^ This finding may therefore have been related to possible differences in the clientele between the two sites regarding socioeconomic conditions and inequalities of access to healthcare services.

In our study, most of the infections were produced by protozoa (13.5%). These results can be explained by the fact that the great majority of the constituents of the sampled group were adults, with an average age between 29 and 42 years. Helminth infections are more frequent in childhood^23^ and may give rise to resistance to reinfection in adults. Another hypothesis explaining why most of the infection were protozoal is that massive screening for helminths and treatment with anthelmintic drugs have been implemented, as part of healthcare programs.^24^ However, indiscriminate use of anthelmintic drugs is a matter of concern. This practice is rooted in popular culture in Brazil, regardless of the social or intellectual level of the population, and it has been reported in other studies.^12^^,^^25^

Among the 13.5% of the infections caused by protozoa, *Escherichia coli*, *Endolimax nana* and *Giardia lamblia* were the most common species, in accordance with previous studies.^21^^,^^25^ However, the rates of infection in our study were smaller, probably due to differences in the population included. Only 4.2% of the protozoal infections were morphologically identified as *Entamoeba histolytica* or *Entamoeba dispar*. For the same route of infection, these results suggest that the dissemination efficiency of the other protozoa was better. The higher prevalence of the other protozoa may be related to the greater quantities and viability of their cysts, compared with those of *Entamoeba histolytica* or *Entamoeba dispar.*

The prevalence of the *Entamoeba histolytica/Entamoeba dispar* complex was 0.6% in the metropolitan region of Belo Horizonte. Studies conducted in Brazil have reported a range of prevalences of *Entamoeba histolytica* and *Entamoeba dispar* prevalence from 3.8%^4^ to 46.3%,^14^ depending on the study population, socioeconomic level and living conditions. In Minas Gerais, a prevalence of 4.4% was reported in urban slums,^21^ and 14.3% among children and adolescents.^25^ In an urban region in southeastern Brazil, the prevalence was 12.1%.^12^ On the other hand, in regions with poor sanitation, parasites are endemic, and the prevalence of the infection is higher. In a recent study in northeastern Brazil, conducted in an urban slum, the prevalence of *Entamoeba histolytica* and *Entamoeba dispar* among children was 46.3%.^14^

Epidemiological studies have shown that cases of invasive amebiasis are caused by *Entamoeba histolytica* and that *Entamoeba dispar* is not found in intestinal or extraintestinal lesions. However, many people infected with *Entamoeba histolytica* are asymptomatic,^3^^,^^26^ and host factors such as gene expression have a role in invasive amebiasis.^27^ In an urban population of children under five, this type of infection was common and carried the risk of developing into severe cases of invasive amebiasis.^28^ Simultaneous infections by both types of amoebae are often found in endemic areas, and people infected with *Entamoeba dispar* alone may manifest intestinal symptoms and high levels of anti-ameba antibodies, such that unnecessary treatment may occur.^29^^,^^30^

The polymerase chain reaction revealed that the prevalence of *Entamoeba histolytica* is very low, compared with that of *Entamoeba dispar*. Studies from different regions of the world have demonstrated that the frequency of *Entamoeba dispar* infection is higher, except for a few countries around the Pacific rim, where the frequency of infection by *Entamoeba histolytica* was higher.^11^^,^^28^^,^^31^^,^^32^ Our data are in accordance with the findings from other studies conducted in Brazil, which also confirmed that the prevalence of *Entamoeba dispar* was higher.^4^^,^^11^^,^^14^^,^^15^^,^^30^

## CONCLUSION

The prevalence of cases of parasitic infections in Belo Horizonte was 15.0%, and most of them were due to protozoa (13.5%). These were mainly *Escherichia coli* (6.0%), *Endolimax nana* (5.2%) and *Giardia lamblia* (1.2%). Only 0.6% of the samples were infected by *Entamoeba histolytica* or *Entamoeba dispar*, of which *Entamoeba histolytica* accounted for 13.9% of the cases. These results suggest that amebiasis may not be a problem in Belo Horizonte, and that clinicians should consider other causes of gastrointestinal disorders.
